# The College of Nursing (Limited by Guarantee)

**Published:** 1920-12-04

**Authors:** 


### The College of Nursing
### (Limited by Guarantee).

BIRMINGHAM AND THREE COUNTIES CENTRE.

OPEN MEETING FOR NURSES.—On Tuesday, December 7, at 5.30 P.M., in the Lecture Theatre of the General Hospital, Birmingham (by kind permission of the Governors), a representative of the Ministry of Labour will speak on "The Unemployment Insurance Act, 1920."

THE following ladies have been appointed to Queen Alexandra's Military Nursing Service for India in the grade of staff nurse (temporary): Miss Edith Jane Clark, Miss Frances Mary Margaret Huxtable, Miss Eleanor Frances Godwin, Miss Dorothy Filmer, Miss Winifred Pickering Tibbles, Miss Mary Elizabeth Francis, R.R.C., Miss Lillian Maude Trotter, R.R.C., Miss Eleanor Jane Browne, Miss Frances Leslie Arnold, Miss Grace Marie McClemens, Miss Dorothy Irene Harris, Miss Irene Birch, Mrs. Winifred Alice Fennell, Miss Julie Marie Clancy, Miss Muriel Eva Annie Margaret Weatherly.

